# Internal Medicine Program Directors’ Perceptions About Accommodating Residents with Disabilities: A Qualitative Study

**DOI:** 10.1007/s11606-024-08936-y

**Published:** 2024-07-17

**Authors:** Maggie Salinger, Mytien Nguyen, Christopher J. Moreland, Anne N. Thorndike, Lisa M. Meeks

**Affiliations:** 1 Division of General Internal Medicine, Warren Alpert Medical School of Brown University, Providence, RI USA; 2 Yale School of Medicine, New Haven, CT USA; 3 Dell Medical School, University of Texas at Austin, Austin, TX USA; 4 Division of General Internal Medicine, Massachusetts General Hospital, Boston, MA USA; 5 Harvard Medical School, Boston, MA USA; 6 Department of Learning Health Sciences, University of Michigan, Ann Arbor, MI USA; 7 Department of Family Medicine, University of Michigan, Ann Arbor, MI USA

**Keywords:** disability, diversity, graduate medical education

## Abstract

**Background:**

While 26% of US adults are disabled, only 3.1 to 9.3% of practicing physicians report having a disability. Ableism within medical training and practice diminishes physician diversity and wellbeing and contributes to healthcare disparities.

**Objective:**

Explore physician barriers to disability equity and inclusion by examining internal medicine (IM) program directors’ (PD) perspectives about recruiting and accommodating residents with disabilities (RWD).

**Design:**

Qualitative study involving semi-structured virtual interviews (conducted December 2022–September 2023; analyzed through December 2023).

**Participants:**

PDs were recruited via email. Purposive sampling captured program diversity in size, location, and affiliations. Convenience sampling ensured PD diversity by gender, race/ethnicity, and age.

**Approach:**

Coders analyzed thematic and discursive content of interview transcripts to characterize PD perspectives about RWDs and accommodations.

**Key Results:**

Of the 15 programs represented, 4 had ≤ 49 and 8 had ≥ 100 total residents. Three were community-based; the rest had academic affiliations. On average, PDs had 17 (SD 8.2) years in practice. Most (11/15) identified as White race; 8/15 as female; and none as disabled. PDs characterized disability as a source of grit and empathy but also as an intrinsic deficit. They worried RWDs could have unpredictable absences and clinical incompetencies. Perceived accommodation challenges included inexperience, workload distribution, information asymmetry about accommodation needs or options, barriers to disclosure (*e.g.*, discrimination concerns), and insufficient accommodation advertising. Perceived facilitators included advanced planning; clear, publicized processes; and access to expertise (*e.g*., occupational health, ombudsmen).

**Conclusions:**

PDs held contradictory views of RWDs. PD insights revealed opportunities to alleviate PD-RWD information asymmetry in recruitment/accommodation processes, which could help align needs and improve representation and inclusion.

**Visual Abstract:**

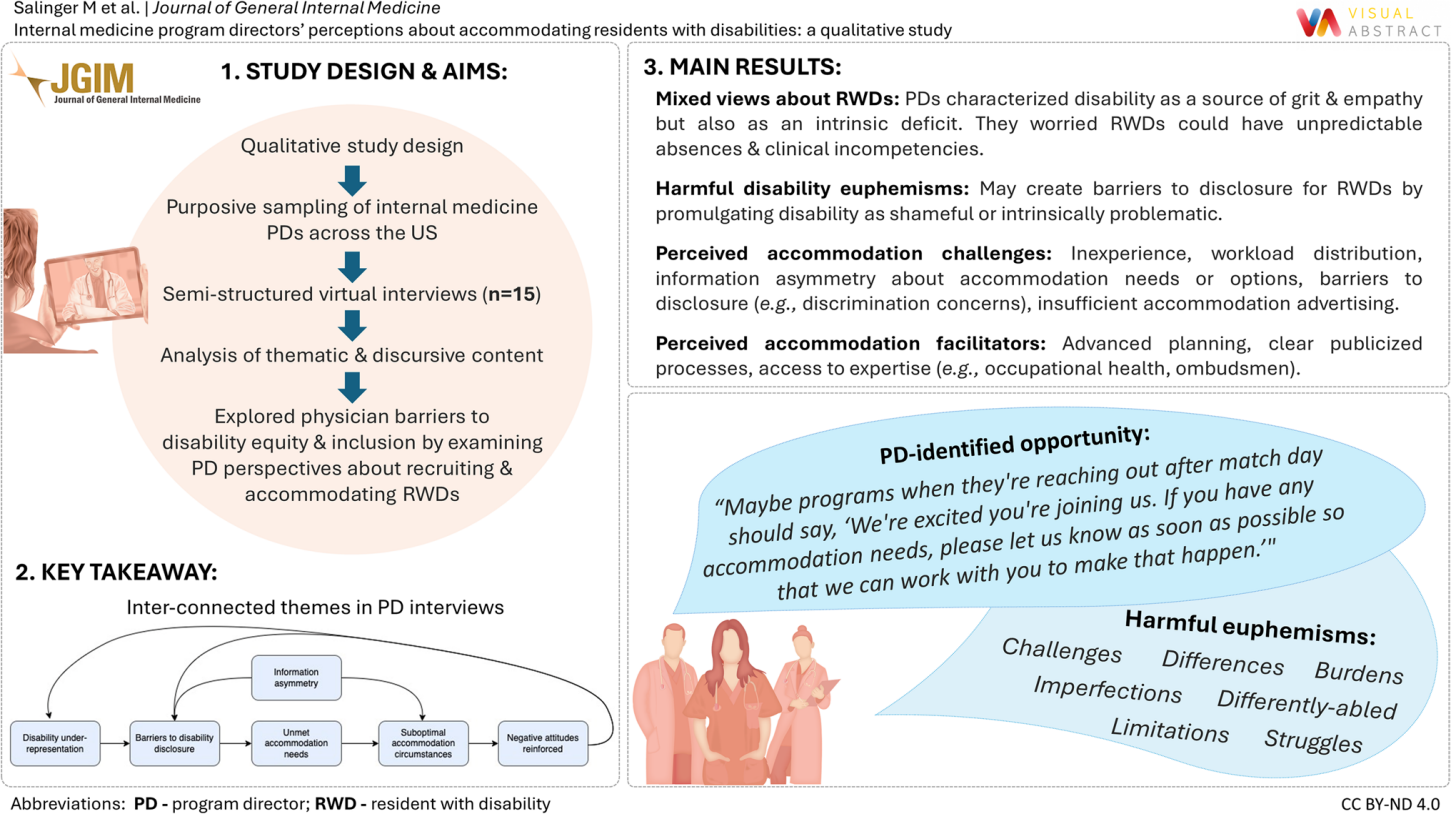

**Supplementary Information:**

The online version contains supplementary material available at 10.1007/s11606-024-08936-y.

## BACKGROUND

People with disability (PWD) constitute the largest minority group in the USA and are affected by health disparities that cannot be explained by symptoms alone.^1–8^ Some disability disparities are linked to medical education and practice, such as clinician-held biases (implicit or explicit) and inadequate training on the fundamentals of caring for PWD.^9–18^ There is a bidirectional relationship between these factors and injustices affecting the physician workforce itself, namely the under-representation of disabled physicians, the mistreatment they experience, and the barriers they face to accessing workplace accommodations.^19–23^

Although the CDC-estimated prevalence of disability among US adults is 26%, the self-reported presence of disability among US physicians ranges from 3.1 to 9.3%.^1,20,22,24^ In a nationally representative survey, physicians with disabilities were more likely to report episodes of physical, verbal, and psychological harm from coworkers or patients when compared to their non-disabled counterparts.^23^

The representation and wellbeing of PWD in the physician workforce are a matter of procedural, distributive, and relational justice — *i.e.*, justice in processes that establish social institutions, in the allocation of resources and opportunities, and in the treatment of people.^25^ *Ableism* can be understood as deficiencies in these arenas or as the social and structural marginalization of PWD.^26^ Examples of ableism range from discriminatory hiring practices to inaccessible features of a workplace environment or false assumptions about a disabled person’s skillset.

The Accreditation Council for Graduate Medical Education (ACGME) requires residency programs to engage in the recruitment, retention, and support of a diverse workforce, also recognizing that these requirements could improve healthcare delivery.^27^ Additionally, the ACGME requires that programs provide accommodations for residents with disabilities (RWD) and maintain a disability-specific policy.^28^ While the details of this ACGME requirement are left to the discretion of individual institutions, disability researchers and advocates have further called for these policies to outline clearly defined protocols for requesting accommodations that do not necessitate self-disclosure to direct supervisors.^19,29^ Prior work has demonstrated incomplete adherence to these specifications and has highlighted related implications for RWD wellbeing and patient care.^30,31^

The importance of workforce diversity extends beyond its potential to alleviate patient care disparities,^32–34^ and while there are ACGME policies that align with justice principles, problems of under-representation, mistreatment, and inadequate accommodations continue to hinder physicians with disabilities. To identify strategies for improving disability equity and graduate medical education (GME) disclosure processes, this study sought to deepen our understanding of internal medicine (IM) residency program directors’ (PD) experiences and perceptions related to recruiting and accommodating RWDs. In doing so, this study also examined the ways in which IM PDs conceptualize disability itself — a construct that lacks a singular definition. After asking each PD for their own definition, the study team proposed one that could be used across interviews, which defined disability as “a limitation in activity, social participation, or opportunity that is caused by a complex interplay of social/structural factors and body functions.”

IM PDs were considered ideal interviewees for several reasons. First, IM is the largest residency specialty, making IM PDs important gatekeepers to clinical practice, including overlap with other specialties through dual, preliminary internship, or transitional year pathways. Second, IM PDs’ perspectives would account for the broad clinical skills and environments in which IM residents train and could therefore identify widely applicable intervention points for mitigating ableism.

## METHODS

This study was reviewed by the Mass General Brigham institutional review board and follows *COREQ* guidelines. Data were collected from December 2022 to September 2023 and analyzed concurrently through December 2023.

### Approach

This qualitative study applied a constructivist paradigm in the design, conduct, and analysis of semi-structured interviews to gain a rich understanding of PDs’ subjective experiences while also considering the broader socio-structural environment of IM residencies.^35,36^ Three study investigators are internists at academic medical centers with an intimate understanding of the general structure, function, and culture of IM GME training. The research topic was conceived by a desire to create equitable opportunities for PWD in medicine. Several team members identify as having a disability and have received accommodations. Since a constructivist paradigm posits that data are co-created by interviewers and interviewees, these personal details are not problematic, but rather help “sensitize researchers to nuances in the data.”^36^

### Recruitment

Recruitment aimed to capture a range of perspectives from PDs (including assistant/associate PDs) of accredited IM residencies across the USA. Doximity Residency Navigator^37^ and FREIDA™^38^ were used to purposively sample by program-level variables, with each sub-category represented at least twice: total residents (*n* < 50, 50 ≤ *n* < 100, *n* ≥ 100), geographic region, metropolitan status, and affiliations (academic institution, community-based, Veterans Health Affairs). Convenience sampling also ensured adequate PD diversity by gender, race, ethnicity, and age.

Participants were recruited and consented via email (Appendix A). “Disability” was not mentioned to avoid selecting for PDs interested in this topic. An attached RedCap questionnaire measured PDs’ demographic traits, GME responsibilities, years in practice, and personal experiences with disability (Appendix B). Recruitment concluded once thematic saturation was achieved.

### Interviews

The interview guide was piloted with a former IM chief resident. All but two PDs conducted the virtual one-on-one interviews on camera. Questions focused on experiences and perceptions of RWD recruitment and accommodations. In keeping with “theory building, rather than theory testing,”^36^ the interview guide (Appendix C) avoided leading questions or thematic assumptions. PDs were asked to share their own definitions of “disability” before hearing the study team’s version.

### Analysis

One team member (MS) conducted and recorded all interviews, which were then transcribed verbatim. Memo-writing helped synthesize findings throughout the data collection and analysis phases. Iteratively, the study team generated a codebook of inductive, deductive, and in vivo codes and then coded each transcript at least twice. Using Dedoose version 9.017 (SocioCultural Research Consultants), coders (MS, MN, LM) analyzed the thematic and discursive content of transcripts to characterize PD perspectives and experiences surrounding RWD recruitment and accommodations. Drawing upon Discourse Analysis and Grounded Theory, thematic analyses derived meaning from multiple factors — such as language and linguistic patterns as well as content, presence, and consistency of messaging — to develop, integrate, and contextualize theories.^35,36,39–42^

## RESULTS

### Interviewee Characteristics

Of the 15 IM programs represented, 4 had ≤ 49 total residents, 3 had 50–99, and 8 had ≥ 100. There were 3 community-based programs, 12 with an academic affiliation, and 10 with a Veterans Health Affairs affiliation. On average, PDs had 17 (SD 8.2) years in practice, and 5/15 were aged ≥ 50 years. Most PDs (8/15) were female,11/15 identified their race as White, and none identified as Hispanic. No PDs reported lived disability experience; 5 had experience through a close friend or family member.

### Disability Perceptions

Supplemental Table 1 outlines themes surrounding PD perceptions of disability. When asked about its meaning, most provided a disclaimer about lacking a working definition. The ensuing responses often characterized disability as an intrinsic deficit and contrasted it with “normal” or “standard” ways of being. Despite this individualistic framing, some PDs did refer to contextual influences, but few mentioned restrictions in social participation, a feature that distinguishes disability from impairments. Throughout interviews, PDs exhibited discomfort with the term “disability,” opting to instead use euphemisms, like “[personal] burdens,” “struggle[s],” “limitations,” “challenges,” “differences,” “imperfect[ions],” or “differently-abled.” One explained that their discomfort stemmed from recognizing external factors, whereas *disability*, to this interviewee, implied something solely situated within an individual:

So, I mean, I think for me, disability is just – I don’t love that term in this because it’s sort of what parts of the job and what ways are you not able to do the job as it is currently constructed…

Similarly, PDs drew comparisons to biopsychosocial conditions, such as depression or addiction, that they emphasized as distinct from disability. Other examples included pregnancy, lactation, parental leave, and family illness. PDs were not probed to justify these distinctions or parallels, even when the described circumstances could fall under the purview of the Americans with Disabilities Act.

Not a disability, but the thing that we encounter the most often where we have to give accommodations… is depression, anxiety, mental health stuff…

PDs perceived disability to be a source of grit and empathy and saw RWDs as opportunities to improve disability awareness and compassion among care teams. They also suggested RWDs could role model vulnerability and perseverance. However, they simultaneously worried about the possibility of RWDs under-performing, lacking competency, having unpredictable absences, and contributing less than their peers.

I had someone [with a disability] matriculate here... And it’s very hard not to have bias – bias isn’t really the word; I think trepidation about the individual’s capacity to graduate and then perform.

### Accommodation Perceptions and Experiences

PDs shared their experiences accommodating residents for not only disabilities and chronic medical conditions, but also pregnancy, lactation, family-related matters, and religious practices. Common examples were changing rotation/call schedules or offering cross-coverage (*e.g.*, to attend appointments or respond to codes). Other accommodations included special diets, allowance of observed (versus performed) procedures, assistive devices and accessible environments, American Sign Language interpreters, closed captioning and auditory aides, visual processing software, and temporary caseload reductions.

Supplemental Table 2 summarizes general attitudes about accommodations. One PD reported that their program’s cultural shift toward becoming more accommodating of all residents had been associated with better performance, increased scholarship, and stronger fellowship matches. Several PDs noted that accommodations or situational “constraints” may inspire broader, systems-level innovations by “forcing change.”

So this isn’t really related to a disability forcing change, but during COVID, because we were short, we actually restructured our entire general medicine service … It was certainly an accommodation made from a constraint. And I’m a big fan of constraints and crises driving positive change.

Conversely, PDs cited potential drawbacks, describing accommodations as “a lot of work,” as a responsibility that “falls on residency leadership,” and as “a strain on the whole system.” Negative attitudes can create conflicts of interest given that the people charged with accommodating RWDs may also recruit and rank prospective trainees. PDs from smaller programs also worried that recommended accommodations for one trainee might result in duty-hour violations for others.

...Luckily, we’ve only have really one resident in my almost 10 years now that needed – the accommodation [recommended by their psychiatrist] was no overnights….And we had a lot of soul-searching about whether or not that was appropriate…We eventually had a hard time complying with that because we were already at the maximum number of nights by our residents…And eventually, that person decided they could do some limited nights, along with their docs.

This excerpt also highlights that RWDs may feel pressured to forego accommodations due to fears of overburdening peers and generating resentment. Concerns about co-worker resentment were common in interviews and are further described in Supplemental Table 2. An oft-cited obstacle to increasing empathy among colleagues was the need to protect RWD privacy.

### Information Asymmetry and Other Accommodation Challenges

Supplemental Table 3 captures perceived challenges of accommodating RWDs, such as knowledge gaps or inexperience with disabled trainees and ill-defined policies and protocols at the institutional or residency program level. Some PDs added that their health system’s office of human resources (HR) lacked “creativity” or familiarity with the GME context, where residents exist in a liminal space, or “purgatory,” between learners and independent practitioners with rotating clinical duties.

I don’t have all the answers...And so sometimes we turn to external resources, go to our ADA office. But oftentimes, those are more regulatory. They’re also not particularly creative. And so, it may take a few iterations to get things right…

**Figure. 1:**
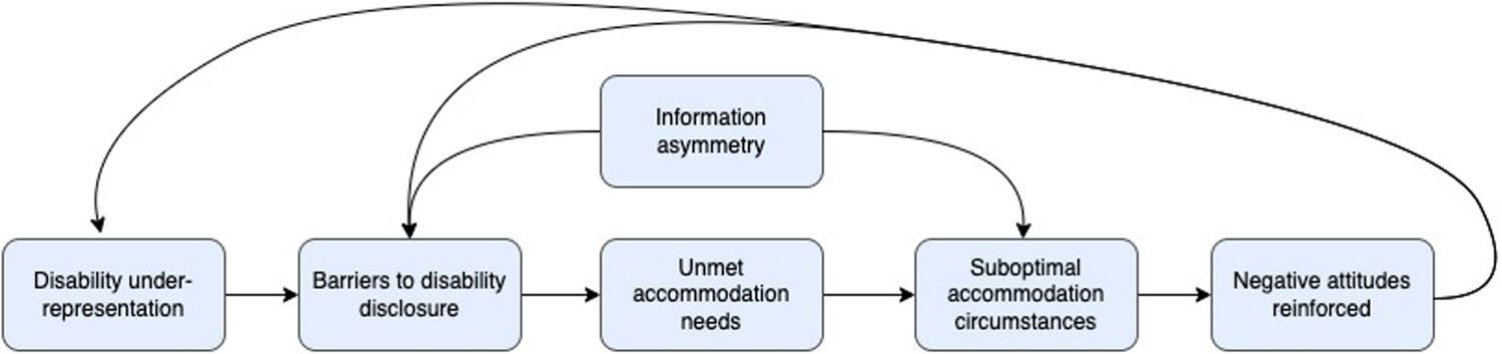
Interconnected themes from interviews with internal medicine program directors (PD) about recruiting and accommodating residents with disabilities (RWD). *Information asymmetry* refers to a knowledge imbalance between program directors and prospective or current residents regarding accommodation needs, options, protocols, and resources. *Disability under-representation* refers to the paucity of physicians with disabilities and the dearth of disability-focused content in residency curricula or diversity programming. *Barriers to disability disclosure* include social factors, such as disability stigma, biases, or discrimination, as well as structural factors, such as disclosure processes that expect RWDs to self-disclose directly to PDs. *Unmet accommodation needs* may result from barriers to disclosure that RWDs encounter. *Suboptimal accommodation circumstances* refers to RWDs having unmet accommodation needs and/or PDs having inadequate access to the resources or time required for arranging accommodations that support RWDs without compromising other programmatic obligations. *Negative attitudes reinforced* refers to concerns or reservations that PDs expressed surrounding RWD recruitment and accommodations.

Remaining challenges are tied to a broader theme of information asymmetry (*i.e.*, a mismatch in type/quality of information) between RWDs and PDs (Fig. 1). While an imbalance of knowledge is inherent in any hiring process, the asymmetry may be accentuated in the residency match, especially with respect to disability and accommodations. For example, several PDs expressed frustration that medical schools accommodate students but do not provide a relevant disability “hand-off” to GME. PDs may feel entitled to this information despite the potential legal obstacles, the inter-institutional conflicts of interest, and the contextual dependence of accommodation needs. A related concern was that accommodations in one stage of medical education could set unrealistic expectations for the next phases of clinical training and independent practice.

… if somebody needed an accommodation plan during medical school, where’s the hand-off or communication of that accommodation plan for medical school to residency or from residency to employment, right? Because it doesn’t help if accommodations that were given to a medical student are completely incompatible with residency…So there also needs to be kind of a shared, I think, understanding across the continuum…

Supplemental Table 3 also demonstrates the difficulty of navigating information asymmetry as PDs try to provide appropriate support to RWDs while maintaining boundaries by not acting as their doctor. The latter was described as particularly complicated when trainees lack established medical care or insight about their own health, especially mental health. To protect RWD privacy, PDs often received limited details about the disability, which was perceived as an obstacle to discerning accommodation needs and devising creative solutions. Considering this tension, PDs noted that some RWDs felt pressured to self-disclose, perhaps to help trouble-shoot or justify their accommodation requests. An additional subtheme was intergenerational resentment, wherein younger physicians were perceived to “have it easier” and have an inflated sense of “entitlement” or “acerbic-ness.”

It’s almost like there’s too much support because then it’s like when do you tease out when someone really needs something versus they’re like, ‘Oh, I think I can get this.’ And you know what I’m saying? So that is a challenge.…I’ve sort of decided from my level down, to the chagrin of some of my APDs, that we’re going to still err on the side of support than not, having some faith and trust in that the residents want to do good.

...I feel like the hardest thing is — our role is not to inquire about their medical or psychiatric illnesses. And so,...it gets a little bit challenging there trying to figure out what their needs are without— I feel like sometimes they feel like they need to tell us things…

Table 1 calls attention to PD-reported causes and consequences of delayed disability disclosure. As phrased by one interviewee, “it usually presents in a crisis,” with performance issues and inadequate time or resources to respond. PDs lamented that prospective RWDs would be justified in fearing discrimination. Since positive traits could be “overshadowed,” PDs conceded they would likely advise that applicants wait until after the match to disclose, even though it would be in both parties’ interest to exchange accommodation information earlier. Yet words like “transparency” and “withhold” suggest feelings of betrayal from postponed disclosure.

…my general advice to applicants would be that transparency is better than not because particularly if this is an issue that you’re going to take with you to residency, then you want to know that you’re going to a program that’s going to embrace you and support you and help you be successful. And so, I think if you would withhold that information, there’s a risk that you end up at a place that doesn’t work for you…though, just not wanting to come across as naïve, if someone has a disability, knowing that they might be discriminated against.

**Table 1:** Program Directors’ Perceptions About the Consequences and Causes of Delays in Disability Disclosure

**Disclosure delays perceived to result in “crisis” and complicate accommodation arrangements**
*“… if we had to create accommodations for somebody that…require a lot of resources, time, money, whatnot, I think that there would probably be a period where kind of be like, okay, well, what do we do? Let’s try to figure out who can we ask questions about this…so there would be a scramble, I think, to just try to figure out what is needed…”* *“Because it usually presents in a crisis rather than with the ability to sort of think ahead and plan… oftentimes, I find these disabilities present during training with performance impairment. And so we’re doing it in the context of high stress of somebody who’s not meeting expectations and not meeting performance goals, and we look to explore why they’re not meeting those expectations or performance goals. And a disability may come to light. And then a trainee trying to grapple with that at the same time we’re trying to come up with a plan is really difficult for a range of reasons, whether it’s the trainee’s own denial about their own conditions, a lack of insight, a lack of a variety of support structures, *etc*.”* *“…Some people are very private about this stuff. And it would only be after they truly have a performance issue that they would come to us.”*
**Fears about disability discrimination in residency recruitment**
*“So I guess on the one hand, [disability] could be seen as a strength or an asset or something favorable, and then on the other, it’s still advised to wait until after [the match to disclose]…You want to be a whole person, right? You want to be a whole applicant, right? …I mean, that’s the biggest [concern], is that just all your other excellent characteristics are g*oing to be totally overshadowed by this, and people are going to view you just very two-dimensionally.”
**Stigma, impostor syndrome, under-representation of disability**
*“And I still think there is, overall, still a stigma associated with those type of [psychiatric] disorders compared to when we have a resident who has a new diagnosis of cancer, I feel like everybody knows about it, right? And everybody’s supportive, and there’s a meal train, and, of course, they’re out of work.”* *“I think there’s a stigma and there has been forever. There’s an expectation that you have to – well, first of all, there is a designation of what "normal" is, right? I did air quotes. And if you are a doctor who has any kind of struggles or any kind of chronic illness or disability, that you will be perceived as not either not being good enough or not being able to perform at whatever the normal standard is. And I mean, we all know that that’s not true, but that’s the perception that is carried down…And then probably if you’re in an institution where you look around and you don’t see exemplars of leaders that are like you, leaders that have disclosed their chronic illness or their disabilities, or see any evidence of a commitment to inclusion of people with disabilities, then that might also lead to a fear of disclosure.”*
**Omission of a disability focus in programs’ diversity, equity, and inclusion efforts**
*“…we focus so much energy on trying to make sure we understand and become inclusive around your identity issues and things like that…but I think we probably don’t spend enough attention on [disability]…I think talking it through makes me realize there are ways to do that much better.”* *“…the main reason for that committee – it’s a resident-run committee—is to kind of create a feeling of belongingness and a home for all residents, but particularly those from more diverse backgrounds. … And so I think integrating into that, again, and making sure that diversity includes disability would be really good…we could probably emphasize that [disability inclusion] if we had a resident who was interested.”*
**Unclear, poorly-publicized protocols for seeking or arranging accommodations**
*“ I think that a huge part of applicants looking and feeling like this is a place where I could belong, where I could feel included, is knowing that there is a solid plan for accommodations beyond ‘tell us if you have accommodations,’ or ‘tell us if you need accommodation.’ And so what we have right now… I think it could be better.”*
**Legal concerns about soliciting accommodation needs from current or prospective residents**
*“…of course, we can’t ask about it – we never do – but I would look upon that really favorably if I was interviewing somebody.”* *“Oh, man. We don’t solicit [information about a disability], and I don’t know if we’re allowed to, because that would be kind of asking a lot about health conditions, which I am not allowed to ask about by state law.”*

PDs understood that, even after matching, stigma and impostor syndrome may impede disclosure and have been fueled by under-representation of disabled role models. Moreover, they acknowledged that their institutions had not included an explicit disability focus in their diversity initiatives, nor had they advertised accommodation availability in residency recruitment. Below, an interviewee lists multiple potential contacts for accommodation inquiries, exemplifying ill-defined processes from an RWD’s perspective:

And so in the interview, when you specifically talk [about potential accommodations] to the program director or somebody in leadership, which hopefully, everybody would have at least one of those conversations or even maybe a coordinator, if the coordinator is very, very involved, then you could get a true sense of what support you would have.

PDs also cited potential legal concerns about soliciting accommodation needs from current or prospective RWDs, stating “we can’t ask about it” and “I don’t think we’re allowed to.”

### PDs’ Recommendations

Finally, Supplemental Table 4 contains PD-identified opportunities to alleviate information asymmetry. Rather than expecting applicants to look past valid disclosure concerns, PDs’ suggestions were to (1) advertise accommodation resources and processes, (2) invite all incoming trainees to make accommodation inquiries, and (3) create opt-out healthcare linkages for all residents. PDs also wanted access to accommodation experts, either within their institutions (*e.g.*, RWDs, GME Ombudsmen or Designated Institutional Officials, HR, and Occupational Health) or by sharing knowledge across programs. They envisioned training workshops and online platforms to exchange insights or ask questions. Another idea was to proactively pilot accommodation types to identify accessibility gaps.

## DISCUSSION

This study is the first to capture IM PDs’ perspectives on recruiting and accommodating RWDs. Interviews revealed interconnected themes with important implications for workforce diversity and equity (Fig. 1). Although PDs had internally conflicting mental models of disability, their insights offered strategies for improving RWD representation and wellness by identifying drivers and alleviators of PD-RWD information asymmetry.

PDs regarded disability as an indicator of perseverance and empathy that enhances patient care but also as a potential liability that could cause scheduling complications, unfair work distributions, or patient safety concerns. Their definitions and attempts to convey RWD support involved euphemisms that inadvertently promulgate disability as shameful or intrinsically problematic.^43^ PDs’ reluctance to use the term “disability” may be tied to both stigma and recognition of the incomplete overlap between disability self-identification versus external assignment of this status.^13,43–45^ Moreover, self-identification preferences vary among members of the disability community regarding person-first versus identity-first terminology.^43^

Framing disability as an intrinsic deficit may create barriers to disclosure and accommodations. In the Intern Health Study, subgroup analyses of RWDs found that half of interns with self-identified accommodation needs did not request them, citing stigma as a primary reason.^22^ The RWDs with unmet accommodation needs had significantly greater increases in depressive symptoms compared to their non-disabled counterparts and were more likely to self-report making major medical errors in comparison to both their non-disabled and their well-accommodated RWD counterparts.^31^ The present study contextualizes these findings by illustrating how stigma-driven barriers to RWD wellness and accommodations reinforce negative perceptions about RWD performance (Fig. 1).

PDs recognized that RWDs’ legitimate fears of stigma and systematic discrimination are exacerbated by inadequate disability representation across the board, from curricular content and diversity efforts to peers and professional role models.^10–16,46^ Yet they hoped RWDs would disclose early to leave ample time for arranging accommodations. Importantly, PD preferences may be unapparent to RWDs, especially applicants who are unfamiliar with residency workflows and programmatic constraints.

PDs identified strategies for reducing information asymmetry by improving access to healthcare and accommodations. Implementation of these strategies would require increased education and confidence in navigating the legal landscape of pre-employment medical evaluations and accommodation solicitations. Programs should also be mindful of conflicts of interest arising from disclosure processes. A survey of family medicine department chairs found that approximately half of their accommodation policies instructed trainees to notify direct supervisors of accommodation needs.^47^

An additional challenge for PDs was determining what would constitute a reasonable accommodation. This was especially true for cases of invisible or non-apparent disabilities that led to uncertainty — even skepticism — about the necessity or appropriateness of requests and that heightened tension around maintaining privacy and professional boundaries.

Considering institutional knowledge gaps, PDs recommended pooling disability resources and sharing accommodation experiences across programs. Platforms for such exchanges should also incorporate RWDs’ perspectives, which PDs endorsed as valuable. They also recommended providing RWDs with opportunities to create disability awareness, which could foster a sense of agency and belonging. Though true, programs must avoid excessive reliance on RWDs to fulfill diversity responsibilities without appropriate recognition or compensation (*i.e.*, the “minority tax”).^48^ Prior qualitative work has illustrated that disabled medical students, residents, and physicians already feel taxed by the level of effort required to obtain or maintain accommodations and that this effort detracts from their professional duties and wellbeing.^21^

### Limitations

Data collection may have been subject to social desirability biases. Despite reaching thematic saturation, this study represents a small sample of IM PDs. Contextual differences across programs and specialties may limit the generalizability of findings.

## CONCLUSION

PDs had contradictory attitudes and beliefs about recruiting and accommodating RWDs. Negative perceptions of RWDs’ and PDs’ use of harmful euphemisms for “disability” (*e.g.*, “struggles” or “imperfections”) may create a reinforcing feedback loop with RWD barriers to timely disclosure. The disclosure barriers that emerged in interviews ranged from stigma and discrimination to inadequate disability representation and poor programmatic marketing of accommodations. Fortunately, PDs identified several opportunities to alleviate PD-RWD information asymmetry, which can improve accommodation access and ultimately RWD equity, inclusion, and wellbeing.

## Supplementary Information

Below is the link to the electronic supplementary material.

Supplementary file1 (DOCX 43 KB)

## Data Availability

The datasets generated and analyzed during this study are not publicly available due to potentially-identifiable information in interview transcripts.
