# Supplementary material for: Internal Medicine Program Directors’ Perceptions About Accommodating Residents with Disabilities: A Qualitative Study
Source: J Gen Intern Med. 2024 Jul 17;40(1):198–206. doi: 10.1007/s11606-024-08936-y (PMC11780235; doi:10.1007/s11606-024-08936-y)
Supplement: Supplementary file 1 — Supplementary file1 (DOCX 43 KB) [file 11606_2024_8936_MOESM1_ESM.docx]

**SUPPLEMENTAL MATERIALS**

1. **Recruitment email**
2. **Demographics questionnaire**
3. **Interview guide**
4. **Supplemental Table 1.** Themes and exemplary quotes surrounding perceptions of *disability* in interviews with internal medicine residency program directors
5. **Supplemental Table 2.** Excerpts from qualitative interviews with internal medicine residency program directors illustrating their general perceptions of workplace accommodations for residents with disabilities
6. **Supplemental Table 3.** Interview excerpts that illustrate program directors’ perceived challenges in the processes of accommodating residents with disabilities
7. **Supplemental Table 4.** Opportunities that program directors identified to improve workplace inclusion and accommodations for residents with disabilities

**Appendix A**

**Recruitment email**

Dear ____,

My name is Maggie Salinger, MD MPP, and I am a GIM fellow at Harvard/MGH. I obtained your email address from [Doximity Residency Navigator; a peer/colleague who is a former resident; a mutual colleague; your institution’s website; FREIDA]. I am hoping to **recruit your residency program to participate in a research study**. The study protocol was reviewed and deemed exempt by my institution’s IRB.

The format is a **1-hour zoom interview (video optional)** that will touch upon the following topics:

1) GME protocols & Covid-inspired changes

2) Workplace accommodations in residency

3) Residency recruitment

By speaking with approximately 20-25 GME-affiliated interviewees across the US, my aim is to learn about the **perspectives and experiences of IM residency program leaders**, such as yourself. *If preferred, you may identify an alternative member of your program’s leadership team to participate* in the interview, such as an associate program director or another colleague with an active GME role and knowledge about the study topics.

**The attached fact sheet** provides further details about this study. If you do agree to participate, then please begin by **completing this brief demographics survey** (1 to 2 minutes). I will then circle back with you to arrange the zoom interview at a time that is most convenient for you.

Thank you so much for your time and consideration.

**Appendix B**

**Demographics Questionnaire**

*(Respondents were permitted to skip questions. However, none did.)*

**· What is your job title with respect to GME?**

**o** Program Director; Assistant Program Director; Associate Program Director; Recruitment Committee; Other core faculty (self-describe)

**· How many years have you been in practice?**

**o** Fill in the blank (numeric values only, max 2 digits)

**· What is your age?**

**o** 20-29 years; 30-39 years; 40-49 years; 50-59 years; >60 years

**· What is your gender?**

o Female; Male; Trans-male; Trans-female; Non-binary; Other

**· Do you identify as Hispanic or Latino?**

o Yes/No

**· Which of the following best describes your race?**

o Asian; Black/African American; White; Native Hawaiian/ Pacific Islander; American Indian/Alaskan Native; Other (self-describe)

**· Do you have any experience with disability, either personally or through close family/friends?**

o Yes, through a close friend or family member

o Yes, through my own lived experience

o No

**· Have you ever received workplace accommodations for a disability or health condition?**

o Yes /No

**Appendix C**

**Interview Guide**

***Reiterating several aspects of informed consent:***

“Thank you for taking the time to do this interview. It will take approximately 1 hour. As a reminder, your participation and your responses will be kept confidential, and the zoom will be recorded to allow for accurate data reporting. Do you have any questions before we begin? Do we have your permission to proceed with the recorded interview?”

***Warm Up***

To begin, can you describe your role and your responsibilities in the residency program?

- e.g., residency recruitment, curriculum design, precepting, didactic teaching,

***Influence of Covid-19 pandemic***

The first few questions are about covid.

In the early, pre-vaccine days of the pandemic, did the program make any adjustments for residents with high-risk medical conditions? If so, what were those changes?

- What factors made it difficult to accommodate trainees with high-risk conditions?

- Was there anything about the covid pandemic or about your institution that helped lower barriers to making these changes (or providing these accommodations)?

Have any trainees in your program been affected by long-covid or sequelae of prior covid illness?

**If yes:**

- How has this impacted their residency training?

- Have they requested or received any special accommodations?

**If no:**

- Would long covid affect a trainee’s ability to fulfill their resident responsibilities?

Has your program leadership thought about what types of accommodations a trainee with long-covid might need?

- How might the residency program respond? How might other trainees respond?

- Are there any potential accommodations needs that seem impractical or infeasible?

***Disability Definition:***

The next set of questions relate to disability and accommodations. Since there are many ways to define disability, can you please share your own version of what it means?

- Do you think the same sort of definition/characterization of disability applies to residents?

Thank you for that. Now I’ll share a definition we can both use for this interview: let’s define disability as a limitation in activity, social participation, or opportunity that is caused by a complex interplay of social/structural factors and body functions.

***Experiences with accommodations:***

What would it be like to have trainees with disabilities or chronic medical conditions in this residency program?

- What are some concerns or challenges? Have any of these concerns played out or posed an issue?

What are some benefits of having trainees with disabilities in the program? What are some ways that the program/institution/profession can elevate or promote these benefits?

What types of accommodations might residents with disabilities or chronic medical conditions need in your residency program?

- What experiences has your program had with providing these types of accommodations?

- What are some challenges about providing workplace accommodations for trainees?

- What would make it easier to accommodate trainees with disabilities or chronic medical conditions?

What would help someone with a disability thrive in your residency program?

- (in addition to accommodations)

- People, policies, or protocols?

- Any “cultural” aspects of the residency program that could make it relatively easier or harder to thrive with a disability?

- What about ways to mitigate or address impostor syndrome?

- Networking opportunities or mentors?

***Disability Perceptions:***

In your opinion, what is the best time for an applicant or incoming resident to disclose a disability?

- Why do you suggest that time (*e.g.,* after the match) as opposed to a different time (*e.g*., before the match.)

- Is there anything that applicants should worry about if they plan to disclose a disability early in the application process?

- How do applicants get information about accommodations in your program?

**Appendix D**

**Supplemental Table 1. Themes and exemplary quotes surrounding perceptions of *disability* in interviews with internal medicine residency program directors**

| **Disability characterized as an intrinsic, individual-level deficit** |
| --- |
| *“Again, I think it's very unique to the individual. I think if there are any differences in which they just have different needs in order to complete the tasks that are required to do.”*  *“I guess compared to a standard, like a standard practicing physician, somebody's unable to perform certain tasks because of a physical or mental ailment.”*  *“…it's almost subjective. But a disability would be something that is a component of your person that is a hindrance to achieving said goal...”* |
| **Contextual factors can affect or mediate disability** |
| *“... it actually depends on what their career goals are and then if that is in alignment with what the program is able to provide in terms of a learning environment.”*  *“...we've picked up on-- things like an attention disorder is perfect, where someone is just so smart they're able to sort of make up for this. And then in residency, they hit the wall because it's just so much harder….”*  *“I think that shows other people that you can struggle at points in your life and still succeed in a career. You just might need to adjust things….and you can still be successful.”* |
| **Discomfort with “disability” and reliance on euphemisms** |
| *“And I think particularly for young doctors who may be naive about what it is like for their patients, to experience life with burdens, that you actually see a colleague who has managed to do it.”*  *“...they are a part of the team who are differently-abled, but they are no less a part of the team than anybody else.”* |
| **Parallel conditions or circumstances that were seen as distinct from disability** |
| *“I mean, and it's not to say that motherhood is a disability, but as an obvious example, whenever I have a resident who is going to be breastfeeding, we talk about what lactation accommodations look like…”*  *“...And not a disability, but an addiction history that was going to require them to do serial monitoring…So it wasn't-- like I said, not a disability, so not to get distracted…”* |
| **Disability as a source of grit or empathy and role modeling vulnerability** |
| *“I might put [disability] in the distance traveled or grit category. And we actually value that quite a bit in our recruitment process.*  *“…I would love the – the self-confidence and the self-efficacy and the disclosure would mean to me that this person is mature and is so forthcoming and that this personal experience will make them a better provider.”*  *“And when you’ve worked with other people who you’ve seen have [disabilities], have kind of had accommodations or worked with that and been very successful…[it] gives that kind of framework, that role modeling that you can reach out for help also.”* |
| **Disability representation as an asset to patient care through enhanced awareness and empathy** |
| *“…so many people don’t know what it’s like to feel slighted or excluded. So I think having that experience, especially very early on, can be so formative, where… you just know how it feels to be different. I think it’d probably make you more compassionate or something.”*  *“I think the community is made stronger by people who are coming from a position where they better understand patients we serve or helping us better understand how to be inclusive of those people.”* |
| **Conflated with under-performance or carrying a smaller share of the workload** |
| *“...it's just you want to put your best foot forward, and I still think for better or worse, this program– or not program, this profession doesn't really have the language to talk about weakness. You know what I mean? So I think a lot of people that go into this profession, they like to do things well, and they like to be looked upon as people that do things well.…You want to fit in kind of thing and be looked at as a contributive colleague. And even if you know that your difference might not make you a bad doctor, I could understand how it might be hard.”* |

**Appendix E**

**Supplemental Table 2. Excerpts from qualitative interviews with internal medicine residency program directors illustrating their general perceptions of workplace accommodations for residents with disabilities (RWD)**

| **Accommodations thought of as opportunities to promote wellness and inspire programmatic change** |
| --- |
| *“…as we've done these things that we've done for people who are disabled or starting families or all the things that are accommodated compared to a traditional residency structure, fellowship applications have gone up, scholarship has gone up, board scores, whatever that's worth, nothing, have gone up.”*  *“One [benefit] is teaching people to care… And so really instilling in that sense of, ‘This is what it means to help someone with accommodations… ‘ [RWDs] force us to reckon with questions we didn’t have to otherwise.…when you have people who are like, ‘Why do we do this? Why am I awake all night?’ When we know that’s bad for all of our brains…And so I think they force us to ask some of these questions that if you just have a young healthy workforce, you don’t ever really have to face.”* |
| **Accommodations seen as a burden for other trainees or program staff (conflict of interest)** |
| *“I’m sure there’s a lot of programs that are like, ‘I don’t want to accommodate because it’s a lot of work, so I’m not going to do it.’ Right?”*  *“[The responsibility of arranging accommodations] falls on the program director, the APD, the chief residents…. So I think all of it really falls on residency leadership, and again, on colleagues…. But I think there's nothing in the healthcare system that supports us to support trainees with disabilities. It is on the residency program to do that.”*  *“When somebody needs a schedule change and you're already in the milieu of residency training… that's hard because your brethren are the ones that carry your workload on their backs…And so if somebody is switching around or something for coverage, it puts a strain on the whole system. And so that is assuredly a challenge.”* |
| **Accommodations regarded as a source or risk-factor for resentment among peers** |
| *“Always worry about stigma. I always worry that people on the surface will be accommodating, but deep in their heart, they may be resentful.”*  *“So I have a resident …who has, at the end of this year, missed…11 weeks of training due to a variety of circumstances... And their colleagues are not happy with them. They have terrible peer evaluations...And I mean, there's nothing I can do about that. And faculty can only fill in so much.”*  *“...if [the residents] perceive that somebody is working less or working in a different capacity, they create stories as to why. And some of them can feel some kind of way… And again, we're smaller programs…I've been shocked in this role as to how many residents know other people's exact number of shifts and weekends and things like that. And again, I can only coach them to not count because it all evens out, but that's on them.”*  *“...one thing that super sucks being a resident is when it's very obvious that things got moved around for you. I mean, not to be mean about it, but that's a really uncomfortable thing for people to be aware.”* |

**Appendix F**

**Supplemental Table 3. Interview excerpts that illustrate program directors’ perceived challenges in the processes of accommodating residents with disabilities**

| **Inexperience and ill-defined protocols; Unique, liminal context of residency training** |
| --- |
| *“A lot of things, like anything in life, is you don't know what you can do until you're faced with it…. But I only know in relation to the specific cases that I've had. And because it's not a large institution, there are not large policies for it.”*  *“In terms of the knowledge base at my institution, I don't think that there is enough education or knowledge amongst all of the GME programs on what to do, how to go about [arranging accommodations].”*  *“And I've been doing this for a long time, and I think I'm doing a fairly good job, but it kind of occurred to me I don't really know what I'm talking about when it comes to advising people what their rights or what accommodations or what policies can be– and the one time we've had to use the ADA, a complaint was made. We used HR…but they may not know a lot about the resident experience and what that really means. Residents are kind of this weird purgatory of trainee/employee, and that's the other challenge, I guess to say, is it's such a weird thing to explain to [HR] people not in this world.”* |
| **Establishing and communicating expectations across training and practice settings** |
| *“…we didn't get a hand-off [from UME] on [a resident’s psychiatric history]. And the person showed up and really had trouble from the second or third day of internship. And it's not that I wouldn't have matched this person, but it would have been much easier had we known. And so we spent a year figuring out why they were having trouble, which turned out to be known to everybody who would know this person before us, and another year or two trying to accommodate those things and help the person succeed…”*  *“…I think medical schools in many ways do a disservice to kids…because it's like they sell them the stars …and then they get to the graduate medical education world and it's like, ‘wow, I was not prepared for the fact that I'm going to have to work on this level.’... They've changed the undergraduate level of expectation. And so certainly, if you've got a disability on top of that and the medical school has moved you through the system, especially because they're putting you on a pedestal because, ‘oh, look, this is diversity, equity, and inclusion’… but they haven't allowed that person to have the insight into what's a realistic practice…”* |
| **Discerning accommodation needs vs. preferences** |
| *“It's a different generation of trainees than when even I was graduating and went through or yourself. And their expectations and their level of entitlement, ‘Well, this is my week off. It should have been protected or whatever,’ is very different. And so then usually people are more than happy and they are gracious, but I've just been surprised by some level of acerbic-ness that I was not expecting from trainees when things like that have to happen. Instead of graciously just being in the support role, oftentimes eventually there's blowback from it.”*  *“One [concern about having RWDs] would be sort of that slippery slope, if you will...the idea of, ’What if everyone starts calling out three days a week?’ …do you drive the culture in a way that is not what we think of as a doctor who's giving themselves to their patients? It becomes too much about the trainee themselves. So I could see that being a sort of theoretical concern.”* |
| **Maintaining boundaries;** **Concerns about residents lacking insight** |
| *“I'm also not anybody's medical provider. So I really have to push trainees to be like, ‘You need a medical provider to make your diagnosis. It is not my role as your program director to diagnose you or to come up with your treatment plan. So I need you to have a medical professional that you are working hand-in-hand with, who can tell me your diagnosis and your recommended management.’ And I find trainees are often very reluctant to disclose that…”*  *“… I found that as long as their insight is significant and they understand what their limitation is, then that is something that can be met…But if you have to fight somebody and say, ‘This isn't safe for patient care for you to do because of X, Y, and Z,’ you can't fix that. So it's really about an individual's level of insight into their own disability....”* |

HR - Human Resources

ADA - Americans with Disabilities Act

UME - Undergraduate Medical Education

APD - Assistant Program Director

**Appendix G**

**Supplemental Table 4. Opportunities that program directors identified to improve workplace inclusion and accommodations for residents with disabilities (RWD)**

| **Boost disability representation and improve accessibility for all** |
| --- |
| *“I think the more people that you have in leadership positions who've gone through residency with disabilities and are in leadership positions, the better…there just aren't a lot of– at least people with known disabilities that are in leadership positions.”*  *“Well, I mean, to see an attending with a disability thriving. I mean, that's a thing, right?…So I think the other thing is, is more visibility of people with visible disabilities might make even someone with a non-visible disability feel a little bit more comfortable.”* |
| **Provide clear, publicized processes for requesting accommodations** |
| *“...I generally try to get people to meet with our HR folks because I feel like that's a little bit out of my comfort zone in terms of what their rights and responsibilities are or have them meet with their ombudsman, which can at least direct them there too, and you lose that stigma of boss versus employee, which is always there. So they have somebody that can be their advocate to help them navigate the system.”* |
| **Leverage lived expertise of RWDs and/or a ‘physical plant’ to identify accessibility gaps** |
| *“… I think, working with the person and saying, ‘You tell me where you might need accommodations,’ but also they were really clear like they do not want to be told where they need accommodations. People want to be a part of that decision-making process.”*  *“...we've had folks…who wanted to be teachers about their process and often give senior grand rounds on what it was like to do what they did with differently abled hearing, vision, physical ability, mental health challenges, things like that. Yeah. So I think feeling like they were an add. You know what I mean? Being like, ‘Yeah. Help us grow. This is not a negative. This is a positive…’”*  *“... until you actually have somebody in your program that does [use a wheelchair], it can be really hard to really understand where the holes are in that. And the number of rooms that don't have automatic doors, the number of rooms that have stairs, so I think that physical plant is another big one that I think is important…”* |
| **Invite accommodation requests and lower barriers to healthcare access** |
| *“I think that connecting to a physician when you're a human of any kind moving to any place, even not in medicine. Finding medical care is extremely difficult. Healthcare is awful, as we all know. But I think that lowering the threshold, lowering the activation energy for finding medical care is one way …We will set you up with a physician, opt out.”*  *“Maybe programs when they're reaching out after match day should say, ‘We're excited you're joining us. If you have any accommodation needs, please let us know as soon as possible so that we can work with you to make that happen.’ And maybe that might take pressure off of the applicant or the newly matched resident…”* |
| **Share resources across programs and institutions** |
| *“That would be the kind of content that I would think would be very helpful in a new program director kind of orientation group or boot camp for somebody who's new to being a PD or APD to do it in a case study format…where there would be a workshop on, ‘Hey, here's a range, or here's some case studies. How would you handle this at your institution and kind of workshop together potential solutions?’”*  *“…[GME] could probably do a better job of…not necessarily having to have every single program have somebody with every single disability come through to learn those lessons, right? So could there be some sort of a national governing body on this? Could we try to learn from one another?”* |
| **Shift to competency-based training, rather than time-based** |
| *“The answer lies in competency-based training. Currently, we do time-based training, right?...there is a great saying, which is, ‘When you make time the constant, everything else becomes variable’ …It's not about how many hours you work or what you can do in a particular day, but are you -- did you meet all the competencies?"* |
| **Heed concerns about one-size-fits-all approaches** |
| “...it would be very helpful to have a list of accommodations to consider for your institution…I think the challenge is, I would be opposed to a list, of saying, ‘These are the accommodations you must offer,’ which is a lot of what the ACGME rules turn into. Because different training settings really are very different…” |

GME - graduate medical education

APD - assistant program director

ACGME - Accreditation Council for Graduate Medical Education
